# Obstinate Vomiting in Pregnancy, with Abortion

**Published:** 1855-10

**Authors:** Jos. P. Logan

**Affiliations:** Atlanta, Ga.


ARTICLE IV.—*Obstinate Vomiting in Pregnancy, with abortion.* BY JOS. P. LOGAN, M. D., Atlanta, Ga.

On the 31st of March 1855, I was called to see Lavinia, a negro woman, aged about 38, the mother of nine children, and found her laboring under great irritability of stomach. Upon investigation, learning that she had passed over the last menstrual period without the return of the usual discharge—it then having been about six weeks since the last flow—I came to the conclusion that she was in all probability, pregnant. I accordingly prescribed some palliative remedies with decided relief to the vomiting for a few days; at the end of which time, however, the previous symptoms returned in an aggravated degree, to which were added, great tenderness in the uterine and gastric regions, the most obstinate constipation of the bowels; and, occasionally the most distressing dyspnea and restlessness.

After protracted efforts to meet the various indications in the case, without being able to give relief, and finding that it was beginning to assume a serious aspect, I requested the advice of my friends, Drs. Darnall and W. F. Westmoreland, in connection with whom, a vaginal examination was made with the speculum, which revealed a prolapsed and enlarged uterus, the mouth being within one inch or less, of the os externum, from which was issuing a small quantity of blood; the lips presented abrasions of their surface which were freely cauterized with the nitrate of silver, and in addition, she was subjected with care, to a varied treatment of bleeding, cupping in the uterine and gastric regions, blistering over the stomach, and a host of remedies internally, with a view of quieting the violent and prolonged vomiting.

Notwithstanding every effort, no beneficial result followed, save the most temporary relief. She being unable to retain anything in the way of food, became greatly reduced, and finally presented alarming Typhoid symptoms.

Having been strongly impressed with the idea, that conception had taken place, and looking to that as the origin of the disturbance, and believing that death was inevitable unless she was speedily relieved; after consultation with Drs. Darnall and Westmoreland in relation to the propriety of inducing abortion, and with their concurrence and the assistance of the latter, a flexible bougie was introduced into the uterus with a view of producing the expulsion of the contents, if pregnancy really existed.

We both used the instrument, passing it up into the uterus about three inches, and after a careful exploration, could discover no conclusive evidence of the existence of anybody within the womb. Thinking it probable that a firmer instrument would be more efficient, I obtained a Surgeon's probe with a bulbous point, well adapted to the purpose, and on the evening of the same day, (the 19th of April,) I introduced this instrument with the same result. She continued with very little change in her symptoms up to the night of the 21st of April, when hemorrhage, with uterine contraction commenced. I was sent for early in the morning and found that she had lost a considerable quantity of blood, I immediately resorted to a vaginal examinatin and found in the vagina a clot of blood, which was brought out, and along with it the product of conception; presenting the usual appearance of a six week's embryo. The uterus had resumed its natural position. Ergot was administered, cold cloths applied, and the hemorrhage was speedily arrested. She steadily and rapidly improved, and in four or five days I considered her out of danger.

The attempt to produce the expulsion of the embryo was not made without a full consideration of the responsibility assumed, and as a last resort, to deliver the woman from impending death—that she would have died, but for the discharge of the ovum, I can scarcely doubt.

We presume it to be unnecessary to enter into a detail of cases or a presentation of authorities to sustain the propriety of the course adopted in this instance. Considering it as we do, an established principle, that in pregnancy, where there is good reason to believe that the mother is about to be sacrificed, as the result of the presence of the fetus in the uterus, it is not only judicious and justifiable, but obligatory practice, to resort to the premature induction of labor.

